# A Review of Complementary and Alternative Medicine Therapies on Muscular Atrophy: A Literature Review of In Vivo/In Vitro Studies

**DOI:** 10.1155/2018/8654719

**Published:** 2018-11-18

**Authors:** Seong-Mok Jeong, Byung-Kwan Seo, Yeon-Cheol Park, Yong-Hyeon Baek

**Affiliations:** Department of Acupuncture & Moxibustion, Kyung Hee University Hospital at Gangdong, 149 Sangil-dong, Gangdong-gu, Seoul 134-727, Republic of Korea

## Abstract

**Objective:**

The objective of this review is to evaluate the recent treatment and study trends of complementary and alternative medicine (CAM) treatments on muscular atrophy by reviewing in vivo/in vitro studies.

**Materials and Methods:**

The searches were conducted via electronic databases including PubMed, the Cochrane Library, China National Knowledge Infrastructure (CNKI), Wanfang MED, and five Korean databases. Only in vivo and in vitro studies were included in this study.

**Results:**

A total of 44 studies (27 in vivo studies, 8 in vitro studies, and 9 in vivo with in vitro) were included. No serious maternal or fetal complications occurred. There were various animal models induced with muscular atrophy through “hindlimb suspension”, “nerve damage”, ‘alcohol or dexamethasone treatment', “diabetes”, “CKD”, “stroke”, “cancer”, “genetic modification”, etc. In 28 of 36 articles measuring muscle mass, CAM significantly increased the mass. Additionally, 10 of them showed significant improvement in muscle function. In most in vitro studies, significant increases in both the diameter of myotubes and muscle cell numbers were reported. The mechanisms of action of protein synthesis, degradation, autophagy, and apoptotic markers were also investigated.

**Conclusions:**

These results demonstrate that CAM could prevent muscular atrophy. Further studies about CAM on muscular atrophy are needed.

## 1. Introduction

Muscle atrophy is defined as a reduction in muscle mass, which is a concept that covers partial or complete wasting away from muscle. In general, functional and morphological changes caused by muscular atrophy usually result in decreased muscle fiber cross-sectional area, protein content, muscle strength, and increased insulin resistance [1]. Muscle atrophy occurs in cachexia, a comorbidity of abnormal conditions such as cancer, AIDS, congestive heart failure, and chronic obstructive pulmonary disease (COPD). It also occurs in sarcopenia, a decrease in muscle mass and strength associated with aging [2].

Although the causes of muscle atrophy are not fully understood, numerous factors contributing to the deterioration of muscle atrophy have been found through recent studies. Among these, decreased alpha motor neuron numbers, increased inflammatory cytokinesis, and decreased hormonal function are considered important factors in its pathogenesis [3].

Meanwhile, according to research by Alfonso et al. [4], the prevalence of sarcopenia in senior citizens aged 60 to 70 years was found to be 5-13% and 50% in patients over 80 years. Around the world, the population over 60 years was estimated to be about 60 million in the year 2000 and has been projected to be about 1.2 billion by 2025 and 2 billion by 2050. On condition that the current average prevalence of muscle loss is maintained, at least 50 million people to date and about 200 million people in the next 40 years will be affected by sarcopenia.

In complementary and alternative medicine, symptoms of muscular atrophy are treated using acupuncture, chuna treatment, and herbal medicine. In addition to these clinical treatments, many studies on complementary and alternative medicine (CAM) therapies for muscular atrophy have been conducted recently, but the results in literature have been insufficient. Against this background, we performed a literature review of CAM on muscular atrophy to establish a research model and suggest a direction for future research.

## 2. Method

### 2.1. Search Strategies

We conducted this literature review of in vivo/in vitro studies through Korean and foreign electronic database searches. Foreign databases used included PubMed, Cochrane Library, China National Knowledge Infrastructure (CNKI), and Wanfang MED. Domestic databases searched included Korean Traditional Knowledge Portal (KTKP), Oriental Medicine Advanced Searching Integrated System (OASIS), Research Information Services (RISS), National Assembly Library, and the Korean studies information Service System (KISS). In the Korean databases, “muscular atrophy”, “muscle loss”, and “complementary and alternative medicine” were used as search terms. In CNKI and Wanfang MED, we used cross-language searches and related search terms which were “muscle atrophy”, “muscle loss”, and “*肌肉減少*”.

The search strategy used in the searches in PubMed and the Cochrane Library was based on a combination of medical subject headings outlined in Table 1. The searches were conducted independently by two investigators (SM Jeong, YC Park) who retrieved relevant studies published before August 2018. After retrieving the articles, we extracted and classified the data by animal or cell models, intervention type (herbal medicine, acupuncture, and moxibustion), and outcome measures (muscle mass, strength, and histological and/or biochemical measurements). The extracted data was then verified by the third author (YH Baek). Disagreements were resolved by consensus through face-to-face discussions among the three authors.

### 2.2. Study Selection

The inclusion criteria for the literature review were as follows: (1) articles on complementary and alternative medicine treatments for muscular atrophy and (2) in vivo or in vitro studies, while the exclusion criteria included the following: (1) clinical trial; (2) articles that were not original; (3) articles that were not in English, Korean, or Chinese; (4) duplicate studies. We also excluded studies that did not focus on muscle loss or muscle cell loss and those which did not use complementary and alternative medicine treatment. Full articles meeting the inclusion criteria were retrieved and read carefully.

## 3. Results

### 3.1. Literature Search

**Figure 1:**
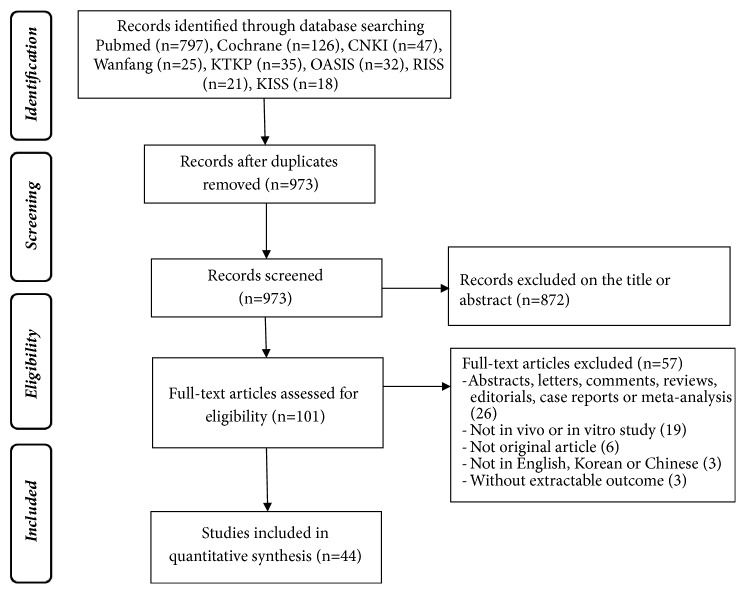
Study flow diagram.

The search initially yielded 973 articles out of which duplicates were eliminated. After screening the titles, 872 articles were excluded due to a lack of adequate coverage of the topic of interest, and after full text assessment, 57 additional articles were excluded for one or more of the following reasons: (1) articles that were not based on original research; (2) articles that did not involve in vivo or in vitro studies; and (3) abstracts and comments. Finally, 44 articles were included in our literature review (Figure 1).

### 3.2. In Vivo Studies

A total of 36 articles (27 of which were in vivo only and 9 of which were in vivo with in vitro studies) were classified as in vivo studies in our review.

#### 3.2.1. Animal Model Species

The most commonly used model was the Sprague-Dawley rat which was used by 22 out of the 36 studies. C57BL/6 mice were used in 6 studies, BALB/c mice were used in 2 studies, and DBA/1 J mice, Kunming mice, and SPF/VAF mice were used in one study each. Three papers did not mention which animal models were used.

There were variations in the ages of the animals used in the studies, with 16-, 12-, 10-, 9-, 8-, 7-, and 6-week-old animals being used in 1, 2, 11, 1, 6, 1, and 7 studies, respectively. In one study, the ages of animals used in the test group differed from that of the control group, being 5 and 19 months, respectively. With regard to the sex of the animals, male rats were predominantly used, featuring in 30 studies, while one study used only female rats and one study used a combination of male and female animals. The other studies did not mention sex of the animals.

**Figure 2:**
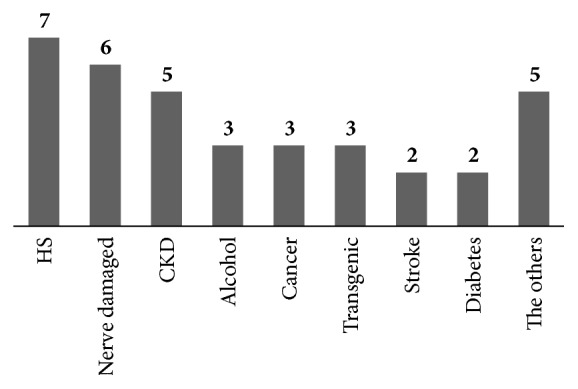
Animal models of in vivo studies.

In order to induce muscular atrophy, various disease models were used in the studies. The most commonly used model for muscular atrophy was the hindlimb suspension (HS) method, developed by Morey-Holton and Wronski [49]. HS rat model was used in 7 of the 36 studies. The next commonly used model was nerve damage-induced muscular atrophy model which was used in 6 studies. Among these studies, direct injury to the sciatic nerve or the peroneal nerve was used to cause atrophy. The chronic kidney disease (CKD) model, in which induction was done by 5/6 nephrectomy was used in 5 studies. The alcohol-induced muscular atrophy model, transgenic model, and the cancer-induced cachexia model (C26-colon-adenocarcinoma implanted) were used, and each of these models was used in three of the 36 studies reviewed. In two studies, diabetes was induced by the administration of streptozotocin to evaluate muscular dystrophy due to diabetes. There were 2 studies using stroke models (middle cerebral artery occlusion, MCAO) and one study for each of the following models: chronic obstructive pulmonary disease (COPD), collagen-induced arthritis (CIA), dexamethasone-induced atrophy, spinal cord injury, and the aging models (Figure 2).

#### 3.2.2. Interventions

**Figure 3:**
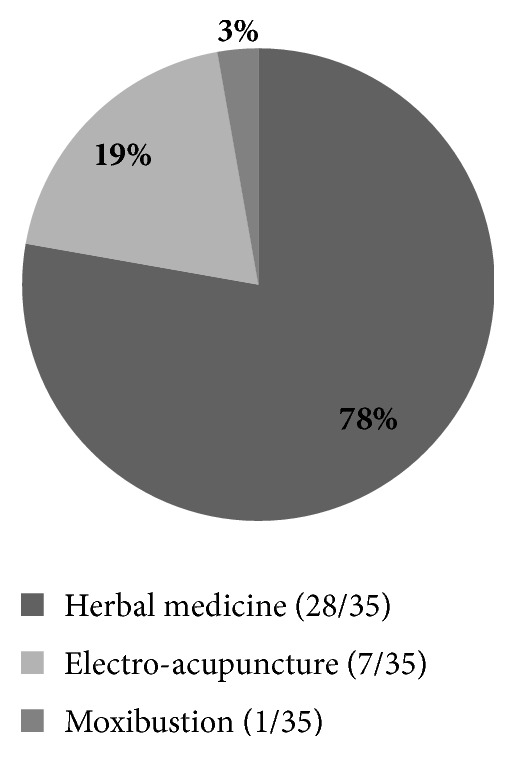
Interventions of in vivo studies.

The most common types of intervention were herbal medicines which were used in 28 studies. In addition, there were 7 studies using electroacupuncture and one study using moxibustion (Figure 3). The treatment periods were 12 days in 2 studies, 2-4 weeks in 24 studies, 1-6 months in 8 studies, and 38 weeks in 1 study. One study did not limit the treatment period.

#### 3.2.3. Outcomes

Muscle mass, the most common primary outcome, was investigated in 28 out of the 36 studies. All except one reported a significant increase in muscle mass. Ten articles investigated muscle function, all of which reported significant increases in muscle strength (Tables 2 and 3).

*(A) Hindlimb Suspension Model*. In the study conducted by Akiko Onda et al., electroacupuncture (E-AT) inhibited the expression of the MuRF-1 gene. E-AT also elevated the expression of Akt1 and TRPV4 [5]. In the study by Zhang ZK et al., icaritin extract was used which inhibited reduced phosphorylation of PI1K-p110, thereby promoting protein synthesis. In addition, the extract inhibited muscle atrophy through modulation of the FoxO3a and FoxO1, the O subclasses of the forkhead family of transcription factors [33]. In the study by Soh KS et al., after using KangwhalSokdan-tang (KWSDT), the ratio of type I muscle fiber/type II fiber was increased showing that KWSDT inhibited early muscular atrophy. IGF-1 and MyoD were also measured and increasing of these key myogenic factors was involved in the improvement of muscle mass [21].

In Cho SG et al., the combination of Schisandra chinensis Baill, Lycium chinense Mill, and Eucommia ulmoides Oliv inhibited muscular atrophy and increased muscle strength and cross-sectional area of muscle fiber [36]. Zhu M et al. also reported the similar effects on muscular atrophy by using Bu Zhong Yi Qi decoction [37].

Bax and Bcl-2 were measured in Kim BH's study. Daeyeoung-jeon extract significantly reduced the immunoreactivity of Bax and increased Bcl-2 in gastrocnemius muscle [23]. In another study by Kim BH, Dangguibohyul-tang (Dangguibuxuetang) extract significantly enhanced the antioxidant enzyme, Cu/Zn-SOD which inhibited disuse muscular atrophy by suppressing oxidative damage [24].

*(B) Nerve Damaged Model*. In the studies using nerve damaged model, one study provided treatment with Radix Puerariae extract and the other used Radix Dipsaci extract. Both studies measured Bcl-2 and Bax. While Radix Puerariae extract was found to significantly inhibit Bax but not Bcl-2 [15], Radix Dipsaci extract significantly inhibited both Bax and Bcl-2 expression [17].

In the study by Zhou L. et al., the result indicated that Buyang Huanwu Tang (BYHWT) decreased inflammatory cells through the activation of angiopoietin-like protein 4 (ANGPTL4) and inhibited muscle cell apoptosis in the denervated-dependent skeletal muscle atrophy model. The effect of BYHWT was mediated by increased expression of NF-*κ*B and MuRF-1 [27]. Another study by Zhou L. et al., BYHWT increased the NF-*κ*B p65 and MuRF1, too [30].

Yu J. et al. applied E-AT on penicillin injection-induced sciatic nerve injury model and the treatment alleviated muscular atrophy by upregulating agrin and acetylcholine receptor-*ε* (AChR-*ε*), and downregulating AChR-*γ* associated in neuromuscular transmission [28]. In Cao R. et al., E-AT on sciatic nerve injury inhibited muscle cell apoptosis by suppressing Bax, cytochrome C, and caspase-3 [31].

*(C) Stroke-Induced Model*. In the studies by Lee et al. and Han et al., the Radix Dipsaci extract inhibited the “slow-to-fast shift" phenomenon in muscle fibers and MHC-II expression while increasing MyoD expression and thereby significant efficacy in muscle recovery [18, 19].

*(D) Diabetes-Induced Model*. Insulin-like growth factor 1 (IGF-1) was the main factor measured in both studies using diabetes-induced model. In the study by Su Z et al., low-frequency electroacupuncture stimulation (LFES) promoted IGF-1 [6]. Additionally, LFES increased Pax7 genes, MyoD, myogenin, and eMyHC. In a study by Zhang J. et al., the Zhimu-Huangbai (1:1) herb pair was used, which promoted IGF-1 and inactivated FOXO3 protein [14].

*(E) Chronic Kidney Disease-Induced Model*. In a study by Hu L. et al., LFES promoted the IGF-1 signaling pathway by decreasing microRNA-1 and microRNA-206 which suppressed IGF-1 transcription [10]. In Lu L. et al., astragalus polysaccharide (APS) reduced proinflammatory cytokines and regulated oxidative stress, thereby inhibiting muscle loss [35].

In Wang D. et al., the administration of Jian-Pi-Yi-Shen (JPYS) decoction prevented muscle loss, muscle protein degradation, and increased muscle protein synthesis. In addition, JPYS decoction not only inhibited FoxO3a activation and ubiquitin-proteasome system (UPS), but also increased Cox IV, NRF-1, and PGC-1*α* [25]. Atrogin-1 and MuRF-1 were also measured in the study by Geng Z et al., and astragalus polysaccharide significantly reduced the expression of atrogin-1 and ubiquitin in rat skeletal muscle [38].

In CKD-induced mice, the levels of autophagy-related proteins including Bnip3 and Beclin-1 were increased. Likewise, the ratio of the autophagy proteins LC3II-to-LC3I, associated with autophagosome formation, was increased. Su Z. et al. suggested that using Acu/LFES suppressed the CKD-induced upregulation of autophagy by inhibiting those proteins [26].

*(F) Tumor-Induced Model*. All the tumor model studies measured markers of the immune system, such as TNF-*α* and interleukins. In the study by Kim AY et al., Sosiho-tang suppressed the production of cachexia-inflammatory cytokines (IL-6, IL-1, and TNF-*α*) and these inhibited the activation of nitric oxide and p38, NF-*κ*B, and STAT3, thereby reducing muscle loss [32]. Another study by Kim AY et al., a novel herbal formula, SGE, also decreased inflammatory cytokines [39] and Zhuang et al. found that the Zhimu-Huangbai (1:1) herb pair reduced tumor-induced muscle loss and inhibited cachectic cytokines [8].

*(G) Dexamethasone or Alcohol-Induced Model*. Kim et al. found a statistically significant inhibition of muscle loss in the dexamethasone-induced muscle atrophy model after treating with Fructus Schisandrae by the activation of SOD, CAT, and GSH [13]. Furthermore, Kim et al. treated with Radix Pueraria extract and Shihosogan-san (SHSGS), both of which inhibited muscular atrophy by inhibiting Bax and promoting Bcl-2 [20, 22].

*(H) The Others*. In a study by Yuki Kishida et al., Go-sha-jinki-Gan (GJG) was administered to a senescence-accelerated mouse which promoted pAMPK and PGC-1*α*, thereby activating mitochondrial neogenesis and oxidative metabolism [9].

Yuya Nakashima et al. demonstrated that significant decreases in muscle loss and oxidative stress due to the reduction of malondialdehyde (MDA) and 4-hydroxyalkenals (HAE) of muscle cells occurred after treating with Chlorella extract [11].

In Zhang YT et al., spinal cord injury-induced (SCI) muscular atrophy model was treated with E-AT [29]. E-AT increased the expression levels of choline acetyltransferase (ChAT), which is a transferase enzyme responsible for the synthesis of the excitatory neurotransmitter acetylcholine [50]. Likewise, E-AT increased the protein neurotrophin-3 (NT-3), a member of the neurotrophin family, and has an important role in neuroprotection and axonal regeneration [51]. Through these mechanisms, E-AT on SCI rat protected the motor neurons as well as alleviated muscular atrophy.

In Tseng YT et al. using spinal muscular atrophy rat model, Liuwei dihuang water extract (LWDH-WE) improved muscle strength and body weight accompanied by upregulation of survival motor neuron protein in spinal cord and gastrocnemius muscle tissues [40].

### 3.3. In Vitro Studies

A total of 17 (8 in vitro only, 9 in vivo with in vitro) articles were classified as in vitro studies in this review.

#### 3.3.1. Muscle Cell Model

**Figure 4:**
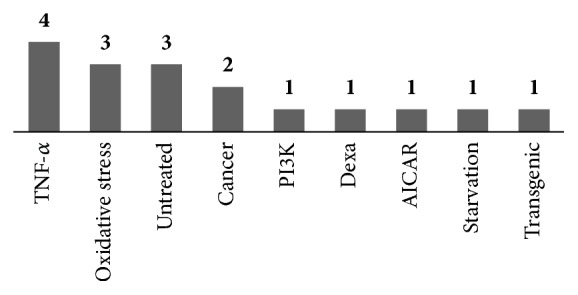
Muscle cell models of in vitro studies.

C2C12 cells (myoblasts and myotubes) were used in 15 of the 17 studies. There was 1 study using L6 myoblasts and 1 using NSC34 motor neuron-like cells. The TNF-*α*-induced apoptosis and autophagy model were used in 4 studies, while the oxidative stress model was used in 3 studies. In addition to these, 3 studies utilized the no treatment model, 2 using cancer model. The dexamethasone-treated, PI3K-p110-suppressed, AICAR-treated, starvation-induced, and transgenic models were used in 1 study each (Figure 4).

#### 3.3.2. Interventions

All 17 studies used herbal medicine as the intervention method. The treatment period spanned 24 hours in 8 studies, 48 hours in 7 studies, and 72 hours in 1 study. One study did not report the treatment period.

#### 3.3.3. Outcomes

Myotube diameter and muscle cell number were set as the primary outcomes. In most studies, significant increases in both the diameter of myotubes and muscle cell numbers were reported (Tables 3 and 4).

*(A) Oxidative Stress Model*. In the three studies using the oxidative stress model, Jung et al. found that idesolide extract protected muscle cells from oxidative stress by promoting the activation of heat shock protein 70 (HSP70) in the cells [42]. HSP70 protects cells from a number of apoptotic stimuli, including oxidative stress and is significantly downregulated in various models of skeletal muscle atrophy [52]. They also found the same effects when sauchinone (root of* Saururus chinensis*) was used as the mode of treatment [44].

In Choe YH., isorhamnetin (ISO) extract was treated to hydrogen peroxide- (H2O2-) induced oxidation damaged muscle cells. In general, H2O2 causes cell apoptosis with mitochondrial damage and ROS generation by upregulation of Bax and downregulation of Bcl-2 [53]. The ISO extract inhibited the apoptosis by the restoration of Bax, Bcl-2 proteins, and decreasing of caspase-9 and caspase-3. In addition, the ISO activated the antioxidant protein heme oxygenase-1 (HO-1) and its transcription factor, nuclear factor erythroid derived 2-related factor 2 (Nrf2), thereby suppressing oxidative stress [47].

*(B) AICAR-Induced Model*. Kang et al. found that the administration of Schisandrae Fructus (SF) extract to AICAR (5-aminoimidazole-4-carboxamide-ribonucleotide)-induced muscle atrophy C2C12 myotubes significantly counteracted AICAR-induced muscle cell atrophy and reversed the increased expression of MuRF-1 and FoxO3a [46]. AICAR is an enzyme that upregulates MuRF-1 and stimulates the levels of the FoxO3a transcription factor [54]. SF extract could reverse the muscle cell atrophy caused by AICAR through regulation of the AMPK and FoxO3a signaling pathways, followed by inhibition of MuRF-1.

*(C) TNF-α-Induced Model*. In Lu L. et al., the ratio of phosphorylated mTOR to total mTOR was decreased, whereas the ratio of LC3B-II to LC3B-I increased after TNF-*α* treatment. APS restored the downregulation of p-mTOR and increased the ratio of LC3B-II to LC3B-I [35]. In Chen D. et al. using Emodin (ED) to TNF-*α* treated C2C12 myoblasts, ED significantly attenuated mitochondrial signaling pathways related cell apoptosis by increasing of Bcl-2 and decreasing of Bax, cleaved caspase-3 and cleaved-PARP. In addition, ED inhibited autophagy by suppressing the expression of LC3-II, Beclin-1, and Atg7 [45].

In the study by Cho SG et al., herbal formula consisting of Schisandra chinensis Baill, Lycium chinense Mill, and Eucommiaulmoides Oliv significantly inhibited protein degradation by decreasing atrogin-1 and MuRF-1 and promoted synthesis by stimulating Akt/mTOR signaling pathway. The formula also increased MyoD and myogenin [36]. Geng Z et al. used APS and pyrrolidine dithiocarbamate (PDTC) to TNF-*α* treated L6 myoblasts. Both extracts delayed muscle cell atrophy through reducing atrogin-1 and ubiquitin [38].

*(D) Untreated Model*. In the studies using the untreated C2C12 myoblast model, Takeda et al. showed that administration of Hachimijiogan (HJG) to untreated C2C12 cells promoted muscle cell proliferation through activation of ERK1/2 signaling pathway without affecting the Akt signaling pathway [43]. In a study by Sung BK et al., the expression levels of myogenic proteins (MyoD and myogenin) and functional myosin heavy chain (MyHC) were measured to evaluate the myogenic potential of loquat leaf extract (LE). LE enhanced those proteins and also activated the PI3K-Akt/mTOR signaling pathway [34]. In Zhu M. et al., the results indicated that Bu Zhong Yi Qi decoction (BZ) significantly downregulated NCoR1 expression and further induced muscle differentiation and metabolism by regulating NCoR1-associated gene expression [37].

*(E) Tumor-Mediated Model*. Kim AY et al. found that sosiho-tang decreased serum IL-6 levels and TNF-*α* [32]. In another study by Kim AY et al., herbal formula, SGE also reduced IL-1*β*, IL-6, and TNF-*α* [39].

*(F) The Others*. In Zhang's study using PI3K-p110-suppressed C2C12 cells, icaritin extract promoted the expression of PI3K-Akt signaling markers [33].

Lu L. et al. reported in a study using the dexamethasone-induced muscle cell injury model that APS increased p-Akt, p-mTOR, p-P70s6k, and p-rsS6 and inhibited FoxO3a/FoxO1 [41].

In Tseng YT et al., inducible survival motor neuron-knockdown NSC34 motor neuron-like cells were used to mimic survival motor neuron- (SMN-) deficient condition. LWDH-WE significantly increased mitochondrial membrane potential and suppressed apoptosis by attenuating the SMN deficiency-induced downregulation of Bcl-2 and upregulation of cytosolic cytochrome c and cleaved caspase-3 [40].

In Li F et al., starvation-induced muscle protein degradation model by incubation of the cells with serum-free basal medium was treated with ginsenoside Rg1. The Rg1 inhibited MuRF-1 and atrogin-1 and also activated the p-mTOR, Akt, p-FoxO1, and p-FoxO3a [48].

## 4. Discussion

As the threat of the global aging phenomenon rises, there has been a growing interest in healthy aging, which in turn has led to an increased interest in muscular atrophy and sarcopenia within the medical community.

Recently, many clinical cases, trials, and preclinical studies have been conducted on the effect of CAM on muscular atrophy; however, a review of the study results and treatment mechanisms reported has been inadequate. We performed the review to establish clinical evidence and suggest a direction for future research in finding treatment mechanisms.

As the results showed, the hindlimb suspension and nerve damaged models were most commonly used in the in vivo studies, which are preferred because they can be induced muscle atrophy by directly and mechanically. CKD model, cancer model, stroke model, and diabetes model were useful for studying muscle atrophy that results from comorbidity of several common diseases.

In many studies, to find the mechanisms of action of muscular atrophy treatment, various markers of muscle protein synthesis and muscular atrophy inhibitors were measured.

In the majority of studies including our review, PI3K-Akt-mTOR signaling pathway factors were investigated. The activation of PI3K leads to activation of the serine/threonine kinase Akt, which in turn phosphorylates and activates its downstream proteins (mTOR, p70S6K, 4EBP1, and eIF4E) and resulted in increased protein synthesis and led to skeletal muscle hypertrophy [55, 56]. Mammalian target of rapamycin (mTOR) which is activated by PI3K-Akt signaling pathway functions as a protein kinase that directly influence cell growth and protein synthesis [57]. As the above markers are included in the major pathways in muscular atrophy, these are suggested to be measured in the related studies.

In addition, other cell proliferation pathway proteins like TRPV4, ERK1/2 could be investigated. TRPV4, a member of the TRP channel superfamily, is known to promote physiological functions in muscle [58], and ERK1/2 is member of the mitogen-activated protein kinase super family that can mediate cell proliferation and apoptosis in the Ras–Raf–MEK–ERK signaling cascade [59].

MuRF1 and FoxO3/atrogin-1 are proteins leading to upregulation of the UPS pathway to catalyze the myofibrillar proteins [60]. FoxO3 can be phosphorylated by Akt, and deactivation of Akt would lead to relocation of FoxO into the nucleus and activate atrophy-induced genes. Activated FoxO3 stimulates lysosomal proteolysis in muscle by activating the autophagy process by transcriptional regulation [61]. So, these proteins are needed to find the activation of UPS pathway in muscle atrophy.

In the cell apoptotic markers, Bax, a proapoptotic factor that promotes cell death, and Bcl-2, an antiapoptotic factor, were mainly used. Caspase-9 and caspase-3 were also measured because they were the key caspase proteases in the activation of intrinsic apoptosis pathway [62]. Through the measurement of those apoptotic factors, the efficacy of suppressing muscle cell apoptosis can be verified.

MyoD, MHC-II, MyHC, and myogenin were measured to investigate the level of myogenesis. MyoD, MHC-II, and MyHC are proteins that play a major role in regulating muscle differentiation, and myogenin is a transcription factor involved in coordination of skeletal muscle development or myogenesis and repair.

Mitochondria related proteins like PGC-1*α*, Cox IV, and NRF-1 were measured to investigate the effect of skeletal muscle metabolism through modulating mitochondrial biogenesis. Also, NCoR1 were measured as a key modulator of the mitochondrial metabolism in muscle, which can reprogram muscle metabolism from a glycolytic to a more oxidative mitochondrial state [63].

In this literature review, we searched for articles on CAM as a treatment modality for muscular atrophy. However, owing to the small number of studies used in the review, only a qualitative analysis of the data could be done. Further studies must be carried out to provide additional evidence to support our findings.

## 5. Conclusion

In this study, outcome measures of muscle mass and function were used to evaluate the effects of complementary and alternative medicine on muscular atrophy and, in most of studies, the results have proven it to be significantly effective. Markers of muscle protein synthesis and muscle atrophy inhibitors were measured to find their treatment mechanisms. In those mechanisms, (1) changes in growth hormone/IGF productivity and sensitivity by aging, (2) decrease of muscle protein by proinflammatory cytokines (TNF-*α*, IL-1, IL-6, etc.), (3) acceleration of apoptosis in muscle cells, (4) ubiquitin-proteasome proteolytic pathway, and (5) Akt/mTOR signaling pathway were found to be important factors contributing to treatment effects.

In most of the studies we have investigated, CAM promoted protein synthesis and inhibited degradation by enhancing the PI3K-Akt-mTOR signaling pathway. It also inhibited the ubiquitin-proteasome system which was involved in protein degradation. In several studies, CAM alleviated skeletal muscle metabolism through modulating mitochondrial biogenesis factors, thereby preventing muscle loss. In addition, CAM restored the expression of proteins involved in the cell apoptotic pathway and autophagy pathway in the various models of muscular atrophy.

## Figures and Tables

**Table 1:** Search strategy.

#1	Muscular atrophy

#2	Muscle mass loss

#3	Muscle weight loss

#4	Muscle wasting

#5	Sarcopenia

#6	#1 or #2 or #3 or #4 or #5

#7	Acupuncture^*∗*^

#8	Acupoint

#9	Herbal^*∗*^

#10	Moxibustion

#11	Moxa

#12	Chinese medicine

#13	TCM

#14	Traditional medicine

#15	Kampo medicine

#16	Conservative therapy

#17	Complementary medicine

#18	#7 or #8 or #9 or #10 or #11 or #12 or #13 or #14 or #15 or #16 or #17

#19	#6 and #18

**Table 2:** Summary of articles on complementary and alternative medicine therapies for muscular atrophy *(in vivo)*.

First Author (year) (Ref)	Design	Origin	Group / Sample-Size (n)	Sample Model (Age) (M/F)	Duration	Outcome Measures	Quantity and Type of Intervention	Main outcomes
Onda A 2011 [5]	In vivo	Japan	Total n = 28 control (7) HS (7) HS + MA (7) HS + EA (7)	Hindlimb suspension (HS) rat model (C57BL/6, 8 wk, M)	2 wk	(1) Muscle weight/body weight (2) Cross sectional area (CSA) (3) Muscle-proteolytic genes (4) Protein synthesis markers	MA (manual acupuncture): 30 min on the gastrocnemius, 5 mm insert EA (electro-acupuncture): 30 min on the gastrocnemius, 5 mm insert, 1 Hz, 6.5 mA 1/day for 2wk	(1) Muscle/body weight: HS < HS+MA (7%, p<0.05) HS < HS+EA (10%, p<0.001) (2) CSA: HS > HS+EA (p<0.05) (3) Atrogin-1↓, MuRF1↓ in EA (p<0.01 vs. HS) Atrogin-1↓ in MA (p<0.01 vs. HS) (4) Akt1↑, TRPV4↑ in MA (p<0.05 vs. HS)

Su Z 2015 [6]	In vivo	China	Total n = 48 control (12) LFES (12) Diabetes (12) Diabetes + LFES (12)	Streptozotocin-induced diabetic mouse model (C57BL/6, 8-10 wk, M)	2 wk	(1) Muscle grip function (2) Muscle weight/body weight (3) Muscle regeneration proteins (4) Protein synthesis markers	LFES (Low-frequency electric stimulation): positive point at GB34, negative point at ST36 20 Hz, 1 mA for 15 min 1/day for 2wk	(1) Muscle grip function: DM + LFES > DM (p<0.05) (2) Muscle weight (Soleus, Extensor digitorum longus): DM + LFES > DM (p<0.05) (3) Pax7↑, MyoD↑, myogenin↑, eMyHC↑ (p<0.05 vs. DM) (4) IGF-1↑, p-Akt↑, mTOR↑, p70S6K↑ (p<0.05 vs. DM)

Dong Y 2015 [7]	In vivo	China	Total n =72 Control + Saline (12) Control + BJ (12) Control + AP (12) COPD + Saline (12) COPD + BJ (12) COPD + AP (12)	Klebsiella pneumoniae & cigarette smoke-induced COPD rat model (Sprague-Dawley (SD) rat, 2 mo, M/F)	12 wk	(1) Muscular tension (2) Muscular fatigue index (3) Expressions of Bnip3 and Cyto-C in muscle tissues	BJ: Bufei Jianpi granules (5 g·kg^−1^·d^−1^) AP: Aminophylline (2.3 mg·kg^−1^·d^−1^) Intragastrically inserted	(1) Muscular tension: BJ > Saline, AP (p<0.05) (2) Muscular fatigue: BJ < Saline, AP (P<0.05) (3) Bnip3↓, CytoC↓ (p<0.05 vs. Saline, AP)

Zhuang P 2016 [8]	In vivo	China	Total n = 60 Normal + Water (C) (20) Tumor + Water (M) (20) Tumor + ZBHP (20)	C26-colon- adenocarcinoma-implanted mice (C57BL/6, 4-6 wk, M)	19 d	(1) Muscle weight (2) Muscle function (3) Pro-inflammatory cytokines (4) Atrophy-related genes (5) IGF-1/Akt and autophagy pathway	ZHBP: Zhimu (Rhizoma Anemarrhena) and Huangbai (Cortex Phellodendri) 1:1 extracted with ethanol 104 mg/kg/day, oral intake	(1) Muscle weight: ZHBP > M (p<0.05) (2) Grip-strength: ZHBP > M (p<0.01) (3) TNF-*α*, IL-6: ZHBP < M (p<0.05) IGF-1: ZHBP > M (p<0.01) (4) Atrogin-1↓, MuRF1↓ (p<0.05 vs. M) (5) SRIT1↑, p-Akt↑, FOXO3↓ (p<0.05 vs. M)

Kishida Y 2014 [9]	In vivo	Japan	Total n = 41 SAMP8 + N (10) SAMP8 + GJG (10) SAMR1 + N (10) SAMR1 + GJG (11)	Senescence-accelerated rat model (SAMP8) Senescence-accelerated aging-resistant rat model (SAMR1) (7 wk, M)	38 wk	(1) Muscle fiber size (2) Myofiber balance (fast/slow) (3) Glycogen synthesis proteins (4) Muscle atrophy proteins	GJG: Go-sha-jinki-Gan 4% (w/w) GJG daily for 38 wk N (Control): normal diet	(1) Muscle fiber: P8+N < P8+GJG (p<0.0001) (2) Fast skeletal muscle: P8+N <P8+ GJG (p<0.0001) p-AMPK↑, PGC-1*α*↑ in P8+GJG (3) IGF-1↑, p-Akt↑, p-GSK3*β*↑ (4) p-FoxO4↑, MuRF1↓, TNF-*α*↓

Hu L 2015 [10]	In vivo	China	Total n = 48 Sham (Sham op) (12) Sham-LFES (12) CKD (12) CKD-LFES (12)	5/6-nephrectomized rat model (mice with chronic kidney disease) (n r, n r, n r)	15 d	(1) Muscle mass (soleus & extensor digitorum longus) (2) Grip strength meter (3) Synthesis-related proteins (4) IGF-1 and MGF	LFES: Low-frequency electric Stimulation acupuncture positive point (GB34) negative point (ST36), 20Hz, 1mA 15 min 1/day for 15 d ^*∗*^needles: 0.25 mm (diameter)	(1) Muscle mass: Sham *≒* Sham-LFES CKD < CKD-LFES (p<0.05) (2) Muscle function: CKD < CKD-LFES (p<0.05) (3) p-Akt, mTOR, p70S6K: CKD < CKD-LFES (p<0.05) (4) IGF-1, MGF: CKD < CKD-LFES (p<0.05)

Nakashima Y 2014 [11]	In vivo	Japan	Total n = 16 Control (8) CSD (8)	ALDH2 ^*∗*^2 Tg rat model (selectively decrease ALDH2 activity) (C57BL/6 mice, n r, M)	6 mo	(1) Oxidative stress marker (2) Muscle tissue injury marker (3) Body weight (4) Gastrocnemius cell size (5) Mitochondrial cytochrome C oxidase activity	CSD: Chlorella-supplemented diet 1% for 6 mo Control: fed basic diet	(1) Urinary isoprostane: CSD < Control (after 4 mo, p<0.05) MDA, HAE: CSD < Control (p<0.01) (2) CPK activity: CSD < Control (p<0.05) CKMB activity: CSD < Control (3) Body weight: CSD > Control (after 4 mo, p<0.05) (4) Cell size: CSD > Control (p<0.05) (5) Cyto-C oxidase activity: CSD < Control (p<0.01)

Kim MJ 2012 [12]	In vivo	Korea	Total n = 12 Control (4) CIA (4) CIA+Moxi (4)	Collagen-induced arthritis (CIA) rat model (Inj of bovine type II collagen) (DBA/1J mice, 6 wk, M)	3 wk	(1) Cross-sectional area (2) Phospho-ERK1/2 (3) Myostatin protein (4) IGF-1 mRNA	Moxi: Moxibustion BL23 (shenshu), ST36 (zusnali) on both sides 5 times/day every other day for 3 wk Kangwha-moxi cone 0.025g per 1point	(1) CSA: Control > CIA+Moxi > CIA (p<0.05) (2) phospho-ERK1/2: CIA > CIA+Moxi (3) Myostatin protein: CIA > CIA+Moxi (4) IGF-1: CIA+Moxi > CIA

Kim JW 2015 [13]	In vivo	Korea	Total n = 48 Control (8) Dexa control (8) Dexa + OM (8) Dexa + FS125 (8) Dexa + FS250 (8) Dexa + FS500 (8)	Dexamethasone-induced muscle atrophy mice (SPF/VAF mice, n r, n r)	24 d	(1) Muscle mass (2) Muscle strength (3) Serum biochemistry (4) Antioxidant defense factor (5) mRNA expression	FS: Fructus Schisandrae extract 125, 250, 500mg/kg, 1/day for 24 d oral intake OM: Oxymetholone 50 mg/kg	(1) Muscle mass (a) Body weight: Dexa < FS125 < FS250 < OM < FS500 (p<0.01) (b) Calf, Gastrocnemius thickness: Dexa < FS 125 < FS250 < FS 500 < OM (p<0.01) (2) Muscle strength: Dexa < FS250, FS500 (p<0.01) (3) Serum creatine↓, CK↓, LDH↑ (p<0.01 vs. Control) (4) MDA↓, ROS↓, GSH↓, SOD↓, CAT↓ (5) Atrogin-1↓, MuRF1↓, SIRT1↓, PI3K↑, Akt1↑, A1R↑ (p<0.05 vs. control)

Zhang J 2014 [14]	In vivo	China	Total n = 45 Normal (C) (15) Diabetes (M) (15) Diabetes + ZB (15)	Insulin deficiency rat model by low-dose STZ injection (C57BL/6, 12 wk, M)	6 wk	(1) Muscle weight (2) Muscle function (3) Myofiber CSA (4) IGF1, Akt/mTOR/FoxO3 signal pathways	ZB: Zhimu-Huangbai (1:1) herb pair 0.1ml/10g, 1/day for 6 wk C, M: Normal saline treated	(1) Muscle (quadriceps) weight: ZB > M (p<0.05) (2) Muscle strength: ZB > M (p<0.01) (3) CSA: ZB > M (p<0.05) (4) IGF-1↑, Akt↑, mTOR↑, p-S6K1↑, FoxO3↓ (p<0.05 vs M)

Jang SO 2009 [15]	In vivo	Korea	Total n = 48 Control (24) Sample (PR) (24)	Sciatic nerve-damaged rat model (SD rat, 10 wk, M)	12 d	(1) Muscle weight (2) Cross-sectional area (3) Bax, Bcl-2	PR: Puerariae Radix extract, 25.0mg/100g, 1/day for 12 d oral intake	(1) Muscle weight: Control < Sample (p<0.05) (2) CSA: Control < Sample (p<0.05) (3) Bcl-2↑, Bax↓ (p<0.05)

Kim BH 2016 [16]	In vivo	Korea	Total n = 30 Normal (10) EtOH (10) EtOH + DKEJ (10)	EtOH-treated rat model (SD rat, 10 wk, M)	8 wk	(1) Body weight (2) Muscle weight (3) Cross-sectional area	DKEJ: Daekumeumja 28.0mg/100g, 1/day for 8 wk	(1) Body weight: EtOH < EtOH + DKEJ (p<0.05) (2) Muscle weight: EtOH < EtOH + DKEJ (p<0.05) (3) CSA: EtOH < EtOH + DKEJ (p<0.05)

Cho JH 2008 [17]	In vivo	Korea	Total n = 36 Sham-OP (12) Control (OP) (12) OP + EC (12)	Sciatic nerve-damaged rat model (SD rat, 10 wk, M)	12 d	(1) Muscle weight (2) Cross-sectional area (3) Bax, Bcl-2	EC: Eucommiae Cortex extract 170mg/100g, 1/day for 12d	(1) Muscle weight: Sham-OP > EC > Control (p<0.05) (2) CSA: Sham-OP > EC > Control (p<0.05) (3) Bax↓, Bcl-2↑

Lee CW 2007 [18]	In vivo	Korea	Total n = 24 Sham (8) Control (MCAO) (8) Sample (MCAO + DR) (8)	Middle cerebral artery occlusion (MCAO) stroke rat model (SD rat, 10 wk, M)	25 d	(1) Muscle fiber type (2) Cross-sectional area (3) MHC-II (4) MyoD expression	DR: Dipsaci Radix 184.4 mg/100g, 1/day for 25d, oral intake	(1) Muscle fiber type (type-I): Control < Sample (p<0.01) (2) CSA: not significant (3) MHC-II↓ (4) MyoD: Control < Sample (p<0.01)

Han SW 2008 [19]	In vivo	Korea	Total n = 18 Sham (6) Control (6) Sample (6)	MCA occlusion stroke rat model (SD rat, 10 wk, M)	4 wk	(1) Muscle fiber type (2) Cross-sectional area (3) MyoD expression	DR: Dipsaci Radix 184.4mg/100g, 1/day for 4 wk, oral intake	(1) Muscle fiber type: type-I ↑, type-II ↓ (p<0.05 vs. control) (2) CSA: control < Sample (p<0.05) (3) MyoD↑ (p<0.05 vs. control)

Kim BH 2017 [20]	In vivo	Korea	Total n = 30 Normal (10) EtOH (10) EtOH + PR (10)	Alcohol-induced (EtOH) muscle atrophy rat model (SD rat, 10 wk, M)	4 wk	(1) Muscle weight (2) Cross-sectional area (3) Bcl-2, Bax	PR: Pueraria radix 4.6mg/100g, 1/day for 4 wk, oral intake	(1) Muscle weight: EtOH + PR > EtOH (p<0.05) (2) CSA: EtOH + PR > EtOH (p<0.05) (3) Bcl-2↑, Bax↓ (p<0.05 vs. EtOH)

Soh KS 2009 [21]	In vivo	Korea	Total n = 24 Normal (6) Control (6) HS (6) HS + KS (6)	Hindlimb suspension rat model (SD rat, 6 wk, M)	2 wk	(1) Muscle weight (2) IGF-1 protein (3) Myogenin protein (4) MyoD protein	KS: KangwhalSokdan-tang 1 mg/100g, 3 times/1day orally	(1) Muscle weight: HS < HS+KS (p<0.001) (2) IGF-1: HS < HS+KS (p<0.05, in Type I) (3) Myogenin: not significant (4) MyoD: HS < HS+KS (p<0.05 in Type I)

Kim BH 2016 [22]	In vivo	Korea	Total n = 30 Normal (10) EtOH (10) EtOH + SHSGS (10)	Alcohol-induced (EtOH) muscle atrophy rat model (SD rat, 10 wk, M)	4 wk	(1) Muscle weight (2) CSA (3) Apoptotic factor	SHSGS: Shihosogan-san 31.5 mg/100g, 1/day for 4 wk, orally	(1) Muscle weight: not significant (2) CSA: not significant (3) Bcl-2↑, Bax↓ (p<0.05 vs. EtOH)

Kim BH 2017 [23]	In vivo	Korea	Total n = 20 Control (CON) (10) DYJ (10)	Hindlimb suspension rat model (SD rat, 10 wk, M)	2 wk	(1) Muscle weight (2) CSA (3) Apoptotic factor	DYJ: Daeyeoung-jeon 259.6mg/100g/day orally CON: Saline, orally	(1) Muscle weight: CON < DYJ (p<0.05) (2) CSA: CON < DYJ (p<0.05) (3) Bax↓, Bcl-2↑ (p<0.05 vs. CON)

Kim BH 2017 [24]	In vivo	Korea	Total n = 20 CON (10) DGBHT (10)	Hindlimb suspension rat model (SD rat, 10 wk, M)	2 wk	(1) Muscle weight (2) CSA (3) Malondialdehyde (MDA) (4) Cu/Zn-SOD activation	DGBHT: Dangguibohyul-tang 236.7mg/100g/day orally CON: Saline, orally	(1) Muscle weight: CON < DGBHT (p<0.05) (2) CSA: CON < DGBHT (p<0.01) (3) MDA: no significant difference (4) Cu/Zn-SOD: CON < DGBHT (p<0.05)

Wang D 2017 [25]	In vivo	China	Total n = 30 CON (10) JPYS (10) Sham-OP (10)	5/6 nephrectomized rat model (SD rat, n r ^*∗*^, M)	6 wk	(1) Renal function (2) Muscle weight (3) CSA (4) Protein synthesis and protein degradation (5) Ubiquitin-proteasome system and FoxO3a activation (6) Mitochondrial biogenesis proteins (7) Muscle autophagy/mitophagy pathway (8) Mitochondrial dynamics	JPYS: Jian-Pi-Yi-Shen decoction, 10.89mg/kg/day orally	(1) BUN, Creatinine: CON > JPYS (p<0.001) (2) Muscle weight: CON < JPYS (p<0.05) (3) CSA: CON < JPYS (p<0.05) (4) Protein synthesis↑, degradation↓ (p<0.05 vs CON) (5) Atrogin-1↓, MuRF-1↓, p-FoxO3a↑, FoxO3a↓ (p<0.05 vs. CON) (6) Cox IV↑, NRF-1↑, PGC-1*α*↑ (p<0.05 vs. CON) (7) LC3II/LC3I ratio↓, Beclin-1↓, P62↓, PINK1↓, Parkin protein level↓ (p<0.05 vs. CON) (8) Fis1↓, Mfn2↑ (p<0.01 vs CON), Drp-1↓, OPA-1↑ (p<0.05 vs. CON)

Su Z 2017 [26]	In vivo	China	Total n = 36 Sham-OP (9) Sham+LFES (9) CKD (9) CKD+LFES (9)	5/6 nephrectomized rat model (C57/BL6 mice, 2-4 mo, M)	2 wk	(1) Autophagosome-proteolysis pathway	LFES: positive point-GB34, negative point-ST36 20Hz, 1mA for 15min	(1) Bnip3↓, Beclin-1↓, LC3II/I ratio↓ (p<0.05 vs. CKD)

Zhou L 2018 [27]	In vivo	China	Total n = 56 Sham-OP (8) Model (DSMA) (8) CON-siRNA +BYHWT (8) ANGPTL4-siRNA +BYHWT (8) BYHWT low (8) BYHWT moderate (8) BYHWT high (8)	Denervated-dependent skeletal muscle atrophy (DSMA) rat model (SD rat, 8-10 wk, M)	2 wk	(1) ANGPTL4 in pathogenesis of DSMA (on Anti-inflammatory effect) (2) Muscle cell apoptosis (3) NF-кB and MURF1 expression	BYHWT: Buyang Huanwu Tang (high: solution diluted 2X / moderate: 4X / low: 8X), 2ml every day, orally ^*∗*^Model: DSMA + saline ^*∗*^ANGPTL4: angiopoietin-like protein 4	(1) Inflammatory Cells: Model > ANGPLT4-siRNA+BYHWT > CON-siRNA+BYHWT (p<0.05) (2) Cell apoptosis: Model > BYHWT-low > BYHWT-moderate > BYHWT-high (p<0.01 vs. Model) (3) NF-кB p65, MURF1: Model > BYHWT-low > BYHWT-moderate > BYHWT-high (p<0.05 vs. Model)

Yu J 2017 [28]	In vivo	China	Total n = 54 NOR (6) SNI 1wk, 2wk, 4wk, 6wk (6 each) NOR+EA 4wk, 6wk (6 each) SNI+EA 4wk, 6wk (6 each)	Penicillin injection-induced sciatic nerve injury (SNI) rat model (SD rat, 7-9 wk, M)	2 wk/ 4 wk	(1) Sciatic nerve functional indices (SFI) (2) Muscle weight (3) Muscle fibre CSA (4) mRNA expression of agrin, AChR-*ε* and AChR-*γ*	EA: Electro-acupuncture at GB30 (positive) and ST36 (negative) 5Hz, 2mA for 30min ^*∗*^1 course: alternate days, three times a week for 2 weeks ^*∗*^2 course: alternate days, three times a week for 4 weeks ^*∗*^1w, 2w, 4w, 6w: euthanased at 1,2,4,6weeks	(1) SFI: SNI < SNI+EA (p<0.05) (2) Muscle weight: SNI < SNI+EA (p<0.05) (3) CSA: SNI < SNI+EA (p<0.05) (4) agrin↑, AChR-*ε*↑, AChR-*γ*↓ (p<0.05 vs. SNI)

Zhang YH 2017 [29]	In vivo	China	Total n = 72 SCI (18) TANES (18) EA (18) Sham-OP (18)	Spinal cord transection surgery treated rat model (SD rat, n r, F)	4 wk	(1) Motor neurons of L3, L5 (2) Neurotransmitter synthetase (ChAT) (3) Neurotrophin-3 (NT-3) expression (4) Muscle weight (5) CSA	TANES: Tail nerve electrical stimulation 2.5-8.0kHz(mid), 1-150Hz(low), 100mA, 20min 5times/week for 4weeks EA: electroacupuncture, GV6, GV9, GV2, GV1, ST36, 60Hz for 1.05s and 2Hz for 2.85s, pulse width 0.5ms, 2min, every other day for 4weeks ^*∗*^SCI: spinal nerve injury	(1) Motor neuron L3: SCI < TANES, EA (p<0.05) L5: SCI < TANES, EA (p<0.05) (2) ChAT (choline acetyltransferase): SCI < TANES, EA (p<0.05) (3) NT-3: SCI < TANES, EA (p<0.05) (4) Muscle weight: SCI < TANES, EA (p<0.05) (5) CSA: SCI < TANES, EA (p<0.05)

Zhou L 2017 [30]	In vivo	China	Total n = 60 Sham-OP (10) Model (10) BYHWT-low (10) BYHWT-middle (10) BYHWT-high (10) MCB (10)	Peroneal nerve injury rat model (SD rat, n r, M)	10d / 21d	(1) NF-*κ*B p65, MuRF1 gene expression in 10day (2) NF-*κ*B p65, MuRF1 gene expression in 21day	BYHWT: Buyang Huanwu Tang (i) low: 3g/kg/day (ii) middle: 6g/kg/day (iii) high: 12g/kg/day MCB: Mecobalamin 500*μ*g/day	(1) NF-*κ*B p65: Model > BYHWT-low > B-middle (p<0.05) > MCB > B-high (p<0.01 vs. model) MuRF1: Model > BYHWT-low > B-middle (p<0.05) > B-high > MCB (p<0.01 vs. model) (2) NF-*κ*B p65: Model > BYHWT-low > B-middle (p<0.05) > MCB > B-high (p<0.01 vs. model) MuRF1: Model > BYHWT-low > B-middle (p<0.05) > B-high > MCB (p<0.01 vs. model)

Cao R 2017 [31]	In vivo	China	Total n = 63 Sham-OP Model EA ^*∗*^groups divided into 7days, 14days, 21days each	Sciatic nerve injury rat model (SD rat, 2 mo, M)	7d / 14d / 21d	(1) Muscle cell apoptosis (2) Apoptotic factor	EA: Electro-acupuncture at ST36 and BL57 5Hz, 1.5mA for 10min once a day for 7d, 14d, 21d	(1) Cell apoptosis (7d, 14d, 21d): Model > EA (p<0.05) (2) Bcl-2↑, Bax↓, Cyto-C↓, Caspase-3↓ in 14d, 21d (p<0.05 vs. Model)

^*∗*^n r: not reported.

**Table 3:** Summary of articles on complementary and alternative medicine therapies for muscular atrophy *(in vivo + in vitro)*.

Authors	Design	Origin	Group / Sample Size (n)	Sample Model	Duration	Outcome Measures	Quantity and Type of Intervention	Main Outcomes
Kim AY 2016 [32]	In vivo/ In vitro	Korea	In vivo Total n = 20 No tumor (5) Tumor + Saline (5) Tumor + SO50 (5) Tumor + SO100 (5)	In vivo: CT-26 s.c injection tumor-bearing mice (BALB/c mice, 6 wk, M)	In vivo: 18 d	In vivo: (1) Body weight (2) Muscle weight (3) NO, inflammatory cytokines (4) phos-p38, IкB*α*, IKK*αβ*, STAT3	Sosiho-tang (SO) In vivo: (i) SO: fed 50 or 100 mg/kg, days 10 to 27 after tumor inoculation (ii) Control: fed saline	In vivo: (1) Body weight: Normal > T + SO (100) > T + SO (50) > T + Saline (2) Muscle weight: Normal *≒* T + SO (100) *≒* T+SO (50) > T + Saline (3) NO: dose-dependently suppress (p<0.05 vs. control) IL-6, IL-1*α*, TNF-*α*, IL-1*β*: T + Saline > T + SO (p<0.05) (4) phos-p38↓, IкB*α*↓, IKK*αβ*↓, STAT3↓: T + Saline > T+SO
			In vitro CON SO-0, -50, -100	In vitro: CT-26-mediated C2C12 myoblast	In vitro: 48 h	In vitro: (1) IL-6, TNF-*α* (2) Muscle wasting & myotubes	In vitro: (i) SO powder dissolved in 10% DS to 50 *μ*g/ml, 100 *μ*g/ml for 48 h	In vitro: (1) IL-6, TNF-*α*: T + Saline > T+SO (10) > T+SO (50) >T+SO (100) (p<0.05 vs. control) (2) Muscle wasting↓ Intact myotubes↑

Zhang ZK 2016 [33]	In vivo/ In vitro	China	In vivo: Baseline (10) Age-matched control (10) HS (10) L-ICT + HS (10) H-ICT + HS (10) W (10) L-ICT + W (10) H-ICT + W (10)	In vivo: Rats with or without wortmannin inj. (15 *μ*m/kg/day) for 28 d HS (SD rat, 3 mo, M)	In vivo: 28 d	In vivo: (1) Mass outcome (muscle weight, fiber CSA) (2) Muscle function	ICT: Icaritin In vivo: (i) Low ICT (L-ICT): 80 mg/kg/day (ii) High-ICT (H-ICT): 120 mg/kg/day, oral intake	In vivo: (1) Mass outcome: HS < L-ICT + HS < H-ICT + HS < Baseline (p<0.05) W < L-ICT + W < H-ICT + W < Baseline (p<0.05) (2) Functional outcome: HS < L-ICT + HS < H-ICT + HS < Baseline (p<0.05) W < L-ICT + W < H-ICT + W < Baseline (p<0.05)
			In vitro: CON ICT 5, 10, 20	In vitro: Wortmannin (W) pre-incubated C2C12 cell	In vitro: 24 h	In vitro (1) PI3K/Akt signaling proteins	In vitro: ICT 5 *μ*M or 10 *μ*M or 20 *μ*M for 24 h	In vitro: (1) PI3K-p110, p-Akt, p-mTOR, p70S6K, p-4EBP1: ICT > ICT+W > W (p<0.05) FOXO1, FOXO3a: ICT < ICT+W < W (p<0.05) Atrogin-1, MuRF-1: ICT < ICT + wort < wort (p<0.05) Atrogin-1, MuRF-1: Control > ICT10 > ICT20 (p<0.05 vs. CON)

Sung BK 2015 [34]	In vivo/ In vitro	Korea	In vivo: Young-Control (YC) Young-LE (YL) Old-Control (OC) Old-LE (OL) (n = 4~6 per group)	In vivo: Young SD rat (5 mo, M) Old SD rat (18-19 mo, M)	In vivo: 35 d	In vivo: (1) Muscle mass (2) Muscle function (3) Muscle CK activity	In vivo: Loquat leaf extract (LE) fed 50 mg/kg/day for 35 d	In vivo: (1) Muscle mass: OL > OC (p<0.05) (2) Muscle strength: OL > OC (p<0.05) (3) CK activity: OL > OC (p<0.05)
			In vitro: CON LE 0.25, 0.5, 1.0, 2.5	In vitro: C2C12 myoblasts (untreated)	In vitro: 24 h	In vitro: (1) C2C12 viability (2) Myogenic proteins (3) CK activity (4) Akt/mTOR pathway	In vitro: LE 0.25, 0.5, 1.0, 2.5 *μ*g/ml for 24 h	In vitro: (1) Cell viability: control < LE (2) MyHC, MyoD: control < LE 0.25 < LE 0.5 < LE 1.0 < LE 2.5 (3) CK activity: control < LE 1.0 < LE 2.5 (p<0.01) (4) PI3K, Akt, mTOR: control < LE

Lu L 2016 [35]	In vivo/ In vitro	China	In vivo Total n = 160 Sham OP (SOP) (40) OP (40) OP + APS (40) SOP + APS (40)	In vivo: 5/6 nephrectomized SD rats (SD rat, 6 wk, M)	In vivo: 155 d	In vivo: (1) Body weight (2) Cross-sectional area (3) p-Akt/mTOR, MuRF1/MAFbx and Autophagy signaling (4) Amino acid carriers (5) Pro-inflammatory & Oxidative factor	In vivo: Control: Normal diet, Sham OP APS: Normal diet with 2% Astragalus polysaccharide	In vivo: (1) Body weight: OP < OP+APS < SOP (p<0.05) (2) Mean CSA: OP + APS > OP (p<0.05) (3) p-Akt, mTOR: OP < OP + APS (p<0.05) MuRF1, MUFbx: OP > OP + APS (p<0.05) Atg7, LC3B: OP > OP + APS (p<0.05) (4) SLC38A2, ATF4: OP < OP + APS (p<0.05) (5) TNF-*α*, IL-15, CRP: OP > OP + APS (p<0.05) MDA: OP > OP + APS (p<0.05) SOD: OP < OP + APS (p<0.05)
			In vitro Control TNF-*α* TNF-*α* + APS 0.05, 0.1, 0.2, 0.5, 1, 2	In vitro: TNF-*α* treated C2C12 cell	In vitro: 48 h	In vitro: (1) Myotube diameter (2) Akt/mTOR, ubiquitin proteasome, autophagy signaling	In vitro: APS: 0.05, 0.1, 0.2, 0.5, 1, 2 mg/mL APS 80 ng/mL TNF-*α* treated	In vitro: (1) Myotube diameter: Control < APS (p<0.05) (2) p-mTOR: Control < APS (p<0.05) LC3B-II/LC3B-I ratio: Control > APS (p<0.05)

Cho SG 2018 [36]	In vivo/ In vitro	Korea	In vivo Total n = 48 normal (8) CON (8) SC 20 (8) SC 100 (8) SSLE 20 (8) SSLE 100 (8)	In vivo: Hindlimb suspension rat model (SD rats, 6 wk, M)	In vivo: 21 d	In vivo: (1) Muscle weight (2) Muscle strength (3) CSA	^*∗*^SC: Schisandra chinensis (Turcz.) Baill ^*∗*^LC: Lycium chinense Mill ^*∗*^EU: Eucommia ulmoides Oliv SSLE: 2:1:1-SC:LC:EU herb pair SLE: 1:1:1-SC:LC:EU In vivo: (orally) (i) 20: 20mg/kg (ii) 100: 100mg/kg	In vivo: (1) Muscle weight (a) Gastrocnemius: CON < SC20 < SC100 < SSLE20 < SSLE100 (p<0.001 ver CON) (b) Soleus: CON < SC20 < SC100 (p<0.05) / CON < SSLE20 < SSLE100 (p<0.001) (c) Tibialis ant: CON < SSLE100 (p<0.01) (2) Muscle strength: CON < SC100, SSLE20 (p<0.01), SSLE100 (p<0.001) (3) CSA: CON < SC100, SSLE20, SSLE100 (p<0.001)
			In vitro TNF LC EU SC SLE SSLE	In vitro: TNF-*α* treated C2C12 cell	In vitro: 24 h	In vitro: (1) Myotube diameter (2) Protein synthesis (ubiquitin-proteasome system)	In vitro: 200 *μ*g/ml	In vitro: (1) Myotube diameter: CON < LC, EU, SC, SLE, SSLE (p<0.001) (2) atrogin-1↓ with SC, SLE, SSLE (p<0.001 vs CON) (3) MuRF-1↓ with LC, EU, SLE, SSLE (p<0.001 vs CON) (4) MyoD↑ with LC, EU, SC, SLE, SSLE (p<0.001 vs CON) (5) Myogenin↑ with EU, SC, SLE, SSLE (p<0.001 vs CON) (6) p-Akt ↑, p-mTOR↑ with LC, EU, SC, SLE, SSLE (p<0.01 vs CON)

Zhu M 2017 [37]	In vivo/ In vitro	China	In vivo Total n = 24 CON (8) HS (8) BZ (8)	In vivo: Hindlimb suspension rat model (Kunming mice, 8 wk, M)	In vivo: 21 d	In vivo: (1) Muscle weight (2) CSA (3) Muscls strength	In vivo: BZ: Bu Zhong Yi Qi decoction 5.93mg/g/day orally	In vivo: (1) Muscle weight (a) Gastrocnemius: HS < BZ (p<0.05) (b) Soleus: HS < BZ (p<0.05) (2) CSA: HS < BZ (p<0.05) (3) Muscle strength: HS < BZ (p<0.01)
			In vitro RS BZ	In vitro: C2C12 myoblasts (untreated)	In vitro: n r	In vitro: (1) NCoR1 (nuclear receptor corepressor 1) (2) Myogenesis	In vitro: RS: rat serum BZ: Bu Zhong Yi Qi decoction	In vitro: (1) NCoR1↓ (p<0.001 vs RS) (2) Pax7↑ (p<0.05), Myogenin↑, MyHC↑ (p<0.01)

Geng Z 2017 [38]	In vivo/ In vitro	China	In vivo Total n = 32 NOR (8) CON (8) APS (8) KT (8)	In vivo: 5/6 nephrectomized rat model (SD rat, 7-8 wk, M)	In vivo: 6 wk	In vivo: (1) Atrogin and ubiquitin	APS: Astragalus polysaccharide, 3g/kg/day for 6 weeks, intraperitoneally / in vitro: 15 mg/1 KT: ketosteril (*α*-ketoacid tablets), 1ml/200g/day for 4 weeks, intravenously CON: saline, 3g/kg/day for 6weeks, intraperitoneally NOR: Sham-OP PDTC: pyrrolidine dithiocarbamate 50 *μ*mol/1	In vivo: (1) Atrogin-1: APS < KT < CON (p<0.05 vs CON) Ubiquitin: APS < KT < CON (p<0.05 vs CON)
			In vitro TNF TNF+APS TNF+PDTC	In vitro: TNF-*α* treated rat L6 myoblasts	In vitro: 48 h	In vitro: (1) Atrogin and ubiquitin (2) Muscle cell diameter		In vitro: (1) Atrogin-1↓, Ubiquitin↓ in APS (p<0.05 vs CON) (2) Cell diameter↑ in APS (p<0.05 vs CON) (3) Atrogin-1↓, Ubiquitin↓ in PDTC (p<0.05 vs CON) (4) Cell diameter↑ in PDTC (p<0.05 vs CON)

Kim AY 2018 [39]	In vivo/ In vitro	Korea	In vivo Total n = 15 CON (5) SGE10 (5) SGE50 (5)	In vivo: CT-26 colon carcinoma-implanted mice (BALB/c mice, 6 wk, M)	In vivo: 15 d	In vivo: (1) Body weight (2) Muscle weight (3) IL-6	SGE: herbal cocktail composed of Ginseng Radix alba, Atractylodis Rhizoma alba, and Hoelen In vivo: SGE 10mg/kg/day or 50mg/kg/day for 15days, orally	In vivo: (1) Body weight: CON < SGE10 (p<0.01), SGE50 (p<0.05) (2) Muscle weight: CON < SGE50 (p<0.01) (3) Serum IL-6: CON > SGE10 > SGE50 (p<0.01)
			In vitro SGE 5 SGE 10 SGE 25 SGE 50	In vitro: CT-26-mediated C2C12 myoblast	In vitro: 48 h	In vitro: (1) Inflammatory cytokines (2) NO production and MAPK/NF-*κ*B activation (3) Muscle cell proliferation protein	In vitro: SGE 5, 10, 25, 50*μ*g/mL, incubated for 48h	In vitro: (1) IL-1*β*↓ (5,10,25,50 p<0.01), IL-6↓ (10,25,50 p<0.01), TNF-*α*↓ (25 p<0.05, 50 p<0.01) (2) NO↓ (10,25,50 p<0.01), iNOS↓ (25,50 p<0.01), p-p38↓, p-ERK↓, p-JNK↓, p-I*κ*B*α*↓ (25,50 p<0.01) (3) MyH↑ (10,25,50 p<0.01)

Tseng YT 2017 [40]	In vivo/ In vitro	China	In vivo Total n = 32 WT (wild-type) (8) CON (8) LWDH15 (8) LWDH30 (8)	In vivo: Survival motor neuron (SMN) deficiency-induced transgenic mice model (n r, n r, M)	In vivo: not limited In vitro: 48 h	In vivo: (1) SMN expression in muscle (2) Muscle strength (3) Body weight	LWDH: Liuwei dihuang water extract In vivo: 15mg/kg/day, 30mg/kg/day, orally	In vivo (1) Survival motor neuron expression: CON < LWDH30 (p<0.001) (2) Hindlimb score: CON < LWDH30 (p<0.001) (3) Body weight: WT > LWDH30 (p<0.01) > LWDH15 (p<0.05) > CON
			In vitro CON LWDH 0.01, 0.1, 1, 10	In vitro: NSC34 motor neuron-like cell (transfected to SMN deficiency)		In vitro: (1) Apoptotic-related pathway	In vitro: 0.01, 0.1, 1, 10*μ*g/mL	In vitro (1) Bcl-2↑, cytochrome c↓, cleaved-caspase-3↓

**Table 4:** Summary of articles on complementary and alternative medicine therapies for muscular atrophy *(in vitro)*.

Authors	Design	Origin	Group / Sample-Size (n)	Sample Model	Duration	Outcome Measures	Quantity and Type of Intervention	Main Outcomes
Lu L 2013 [41]	In vitro	China	Control DEX DEX + APS APS	Dexamethasone (DEX) or peroxide-induced atrophy C2C12 cells	24 h	(1) Myotube diameter (2) Akt/mTOR activation (3) PTP1B expression and activity (4) Myoblasts proliferation (5) Anti-apoptosis (6) Apoptosis related protein expression	0.2 mg/mL Astragalus polysaccharide (APS)	(1) Muscle fiber diameter: DEX + APS > DEX (p<0.05) (2) p-Akt↑, p-P70s6k↑, p-mTOR↑, p-rpS6↑ (p<0.05 vs. DEX) (3) PTP1B expression & activity: DEX > DEX + APS (p<0.05) (4) Proliferation rate: 50 *μ*g/mL < 0.1 mg/mL < 0.2 mg/mL (p<0.05 vs. Control (Con)) (5) Apoptotic cells: H2O2 > H2O2 + APS (p<0.05) (6) Caspase-8, Caspase-3: H2O2 > H2O2 + APS Bcl-2: H2O2 < H2O2 + APS (p<0.05) Bax: H2O2 > H2O2 + APS (p<0.05)

Jung MH 2010 [42]	In vitro	Korea	Control H2O2 + IDS 1 *μ*M H2O2 + IDS 5 *μ*M H2O2 + IDS 10 *μ*M H2O2 + IDS 25 *μ*M H2O2 + NAC 5 mм	C2C12 cell-cultured with H2O2 (0.1, 0.5, 1, 2, 4 mм)	24 h	(1) Antioxidant effects (2) HSP70 expression	Pre-treatment with Idesolide: cultured for 24 hr with 1, 5, 10, 25 *μ*м idesolide or without idesolide	(1) Cell viability: Con < IDS 1*μ*м < 25 *μ*м < 5 *μ*м < 10 *μ*м (p<0.001 vs. Con) Con < NAC 5 mм < IDS 25 *μ*м (p<0.001 vs. Con) (2) HSP70: H2O2 < H2O2 + IDS (p<0.001)

Takeda T 2015 [43]	In vitro	Japan	Control HJG 1 *μ*g/mL HJG 10 *μ*g/mL HJG 50 *μ*g/mL HJG 100 *μ*g/mL HJG 200 *μ*g/mL	C2C12 myoblasts (untreated)	3 d	(1) Cell proliferation (2) ERK1/2 phosphorylation (3) Akt activity (4) Differentiation of cells	Hachimijiogan (HJG): 1~200 *μ*g/mL for 3 d	(1) Cell number: HJG (1, 10, 50, 100, 200) > Control (p<0.05) (2) ERK1/2 activity: HJG200 > Control (2.89 fold, p<0.0001) (3) Akt activity: Not significant (4) Cell differentiation: Not significant

Jung MH 2011 [44]	In vitro	Korea	Untreated H2O2 H2O2 + Sau 10 H2O2 + Sau 25 H2O2 + Sau 50 H2O2 + NAC	H2O2 treated C2C12 cell	24 h	(1) Cell viability (2) HSP expression (3) Ceramide content	Sauchinone (Sau): cultured for 24 hr with 10, 25, 50 *μ*M positive control: N-acetyl cysteine (NAC)	(1) Cell viability: H2O2 + Sau > H2O2 + NAC > H2O2 (p<0.01) (2) HSP 70 expression: H2O2 + Sau > H2O2 (p<0.01) (3) Ceramide: H2O2 > H2O2 + Sau (p<0.01)

Chen D 2016 [45]	In vitro	China	Control TNF-*α* TNF-*α* + ED 5 *μ*M TNF-*α* + ED 10 *μ*M TNF-*α* + ED 20 *μ*M	TNF-*α*-induced apoptosis and autophagy C2C12 myoblast	24 h	(1) Anti-apoptosis (2) Mitochondrial pathway (3) Autophagy (4) p-Akt activation	Emodin (ED): component of Rheum palmatum 5, 10, 20 *μ*M	(1) Apoptotic cells: TNF > TNF + ED20 > TNF + ED10 (p<0.05) (2) Bcl-2/Bax↑, cleaved-caspase 3↓, cleaved-PARP↓ (p<0.05) (3) LC3↓, Beclin-1↓, Atg7↓ (p<0.05 vs. TNF) (4) p-Akt↑ (p<0.05 vs. TNF)

Kang YS 2015 [46]	In vitro	Korea	Control SF 100 SF 200 AICAR AICAR + SF100 AICAR + SF200	AICAR-induced muscle atrophy C2C12 myotubes	2 d	(1) Myoblast differentiation (2) Muscle atrophy markers	Schisandrae fructus (SF): Extract of the fruits of Schisandra chinensis Bailon 100, 200 *μ*g/mL	(1) Myosin-heavy-chain↑, Myogenin↑ (2) FoxO3a, MuRF-1. AMPK↓: AICAR > AICAR + SF100 > AICAR + SF200

Choe YH 2015 [47]	In vitro	Korea	Normal H2O2 ISO H2O2 + ISO	H2O2 (1 mM) treated C2C12 cell	24 h	(1) Anti-oxidative stress effect (2) Anti-apoptosis (3) ROS marker (4) Bcl-2, Bax level (5) Caspase activity (6) Nrf2/HO-1	Isorhamnetin (ISO): a flavonoid derived from Hippophae rhamnoides L. 30 *μ*M	(1) Cell viability: H2O2 < H2O2 + ISO (2) Anti-apoptosis: H2O2 < H2O2 + ISO (3) ROS: H2O2 > H2O2 + ISO (inhibit 64% vs. H2O2) (4) Bcl-2↑, Bax↓ (5) Caspase-9, Caspase-3: H2O2 > H2O2 + ISO (p<0.05) (6) Nrf2↑, HO-1↑

Li F 2017 [48]	In vitro	China	CON SF Rg1 10^−4^ Rg1 10^−3^ Rg1 10^−2^ Rg1 10^−1^	Starvation induced muscle protein degradation C2C12 myoblast	48h	(1) Atrogin-1 and MuRF-1 expression (2) PI3K-dependent phosphorylation of AKT, FoxO and mTOR	SF: Serum-free medium for 48h incubation (Starvation induced) Rg1: Ginsenoside Rg1 10^−4^, 10^−3^, 10^−2^ and 10^−1^ mM. incubate for 48h	(1) Atrogin-1: SF > Rg1 10^−1^ > Rg1 10^−4^ > Rg1 10^−3^ > Rg1 10^−2^ (p<0.05 vs. SF) MuRF-1: SF > Rg1 10^−4^ > Rg1 10^−3^ > Rg1 10^−2^ (p<0.05 vs. SF) (2) p-FoxO1↑, p-FoxO3a↑, p-Akt↑, p-mTOR↑ (p<0.05)
